# ID 150 - Oficinas de ATS para os Núcleos de Apoio Técnico ao Judiciário (NatJus)

**DOI:** 10.5327/2237-9622.2025.v34s1.150

**Published:** 2025-11-25

**Authors:** Verônica Colpani, Ana Luiza Cabrera Martimbianco, Camila Monteiro Cruz, Carolina de Oliveira Cruz Latorraca, Cecília Menezes Farinasso, Isabela Porto de Toledo, Patrícia do Carmo Silva Parreira, Rafael Leite Pacheco, Roberta Borges Silva, Rachel Riera

## Abstract

**Introdução:**

Este estudo foi realizado no Nats/NEv do Hospital Sírio-Libanês (HSL) por meio do projeto "Apoio à Decisão Judicial Relacionada à Saúde no Brasil", em colaboração com o Conselho Nacional de Justiça e com financiamento do Ministério da Saúde (Programa de Apoio ao Desenvolvimento Institucional do Sistema Único de Saúde – Proadi-SUS). O estudo teve como objetivo relatar a implementação de uma ação educativa voltada para profissionais de saúde que oferecem suporte técnico-científico a magistrados em decisões judiciais relacionadas à saúde. Foram promovidas oficinas presenciais, com duração de oito horas cada, destinadas a capacitar os profissionais dos Núcleos de Apoio Técnico ao Judiciário (NatJus) na elaboração de sínteses de evidências para casos concretos a serem julgados. Em 2023, os tribunais estaduais sediaram 11 oficinas conduzidas por pesquisadores do HSL especializados em ATS, capacitando um total de 223 participantes. As oficinas foram adaptadas às necessidades específicas de cada NatJus e utilizaram estratégias pedagógicas baseadas em metodologias ativas.

Este trabalho será apresentado oralmente, com apoio de eslaides. A iniciativa pode ser útil para outros países que enfrentam um cenário similar, onde os processos judiciais nesta área geram impacto financeiro significativo e ameaçam a garantia de cuidados de saúde universais e equitativos.

**Método:**

A seguir estão descritas as atividades e os itens/equipamentos necessários para execução: contato inicial com coordenador de cada NatJus para convite de participação nas oficinas – computador/celular para contato via e-mail; solicitação da lista de indicados/convidados da oficina, incluindo magistrados e profissionais dos NatJus, contato via e-mail e envio de formulário desenvolvido no Microsoft Form.

Alinhamento de expectativas do NatJus e magistrados para as oficinas –Formulário desenvolvido no Microsoft Forms.

Credenciamentos dos participantes na plataforma e-NatJus – contato via e-mail e envio de formulário desenvolvido no Microsoft Form.

Avaliação do conhecimento dos participantes pré-oficina – plataforma de aprendizagem interativa, computador/celular.

Oficina – aulas presenciais com atividades expositivas e em grupos, e uso estratégias pedagógicas baseadas em metodologias ativas. – Espaço do evento (sala, auditório etc.) com estações de trabalho, projetor, computador, plataforma de aprendizagem interativa.

Avaliação do conhecimento dos participantes pós-oficina – plataforma de aprendizagem interativa, computador/celular.

Avaliação de satisfação dos participantes – formulário desenvolvido no Microsoft Form.

Confecção dos certificados de presença – computador/celular para contato via e-mail.

